# A Marked Disadvantage: Rapid Urbanization and Mortality of Young Children in Nigeria

**DOI:** 10.1289/ehp.118-a259b

**Published:** 2010-06

**Authors:** Julia R. Barrett

**Affiliations:** **Julia R. Barrett**, MS, ELS, a Madison, WI–based science writer and editor, has written for *EHP* since 1996. She is a member of the National Association of Science Writers and the Board of Editors in the Life Sciences

Individual-level socioeconomic position has long been recognized as a factor in childhood mortality, with lower position carrying a higher risk of death before age 5. Recent research suggests that living in a socioeconomically disadvantaged area increases a child’s risk even after adjusting for factors such as mother’s education or income. A new study using data from Nigeria shows higher rates of under-5 mortality coincided with increased urbanization and uniquely accounts for the impact of disadvantaged neighborhoods on mortality in this age group **[*EHP*** **118:877–883; Antai and Moradi]**.

The pace of urbanization in low- and middle-income countries, paired with inadequate economic performance and other constraints, can result in urban residents increasingly living in areas with overcrowded or deteriorating housing, few social amenities, poor environmental and sanitary conditions, and a lack of economic opportunities. Such conditions are associated with an increased risk of infectious disease and death, with under-5 mortality rates in particular reflecting the degree of socioeconomic development in specific geographic areas.

Nigeria has very rapidly shifted from a mostly rural nation to a heavily urbanized one. In 1970 only 16% of the population lived in an urban area compared with an estimated 40% or more today. The current study used cross-sectional data from the 2003 Nigeria Demographic and Health Survey to assess how urbanization related to under-5 mortality rates and to evaluate the influence of area-level socioeconomics. A subsample of 1,350 mothers and 2,118 of their children, representing 165 administratively defined communities, was selected from the data, which provided demographic and socioeconomic information as well as children’s birth order and time intervals between siblings’ births. Neighborhoods were ranked by “urban area disadvantage index” scores, calculated by the percentage of children living in households without piped water, flush toilets, electricity, or nonpolluting cooking fuel; whose mothers were unemployed or uneducated; and whose households were overcrowded or among the poorest 40%.

Analysis revealed that under-5 mortality increased in the periods 1979–1983 and 1999–2003. Additionally, after controlling for individual child and maternal factors, under-5 mortality rose with urban area disadvantage index score. The researchers concluded that living in a socioeconomically disadvantaged neighborhood independently increased mortality for children under 5 years old. Additionally, they confirmed other research showing that first-born status and short interval between births increased the risk of early childhood death. This study highlights a need for data to better define relationships between urban environments and health, a focus on reducing inequalities, and a promotion of longer birth intervals.

## Figures and Tables

**Figure:**
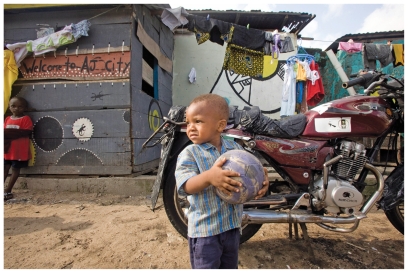
Children play in the Lagos slum of Ajegunle, known locally as AJ City.

